# The Role of ROS‐Mediated Mitochondrial Dysfunction in the Development of Malignant Melanoma

**DOI:** 10.1111/exd.70168

**Published:** 2025-09-24

**Authors:** Lei Li, Zhiwei Zeng, Tengxiao Ma, Bo Hu, Muye Guo, Qiaoxing Wang

**Affiliations:** ^1^ Department of Plastic and Cosmetic Surgery, Hainan General Hospital Hainan Affiliated Hospital of Hainan Medical University Haikou Hainan China

**Keywords:** malignant melanoma, mitochondrial dysfunction, reactive oxygen species, tumour microenvironment

## Abstract

Reactive oxygen species (ROS) are critical to cellular metabolism, signal transduction and apoptosis. Recent studies have revealed the dual role of ROS in malignant melanoma pathogenesis and progression, where they can both promote tumour proliferation and metastasis or inhibit tumour growth by inducing apoptosis. Mitochondria, often referred to as the energy factories of cells, are closely involved in ROS generation, and their dysfunction significantly affects cellular homeostasis. This review explores the mechanisms by which ROS‐mediated mitochondrial dysfunction contributes to malignant melanoma, focusing on its effects within the tumour microenvironment and its potential as a therapeutic target. Understanding these interactions is essential for developing novel strategies to combat malignant melanoma.

## 1 Introduction

### 1.1 Epidemiology and Clinical Features of Malignant Melanoma

Malignant melanoma is one of the most malignant forms of skin cancer, which has been on the increase in different populations. Epidemiological tendencies show that there are notable geographic, age and gender differences. Incidences have been reported in the fair‐skinned people exposed to ultraviolet (UV) rays, and Australia is one of the countries with the greatest rates. On the other hand, malignant incidence is relatively low in places that are exposed to lesser UV rays [1]. Malignant is characterised clinically by the asymmetry, irregular shape, uneven colour and size greater than 6 mm, which are summarised as the ABCDE rule (asymmetry, border, colour, diameter, evolving) [2]. It has powerful metastatic capability that complicates its treatment and medical prognosis. Appreciating these epidemiologic trends and clinical manifestations has importance to establish specific prevention and treatment measures.

**FIGURE 1:**
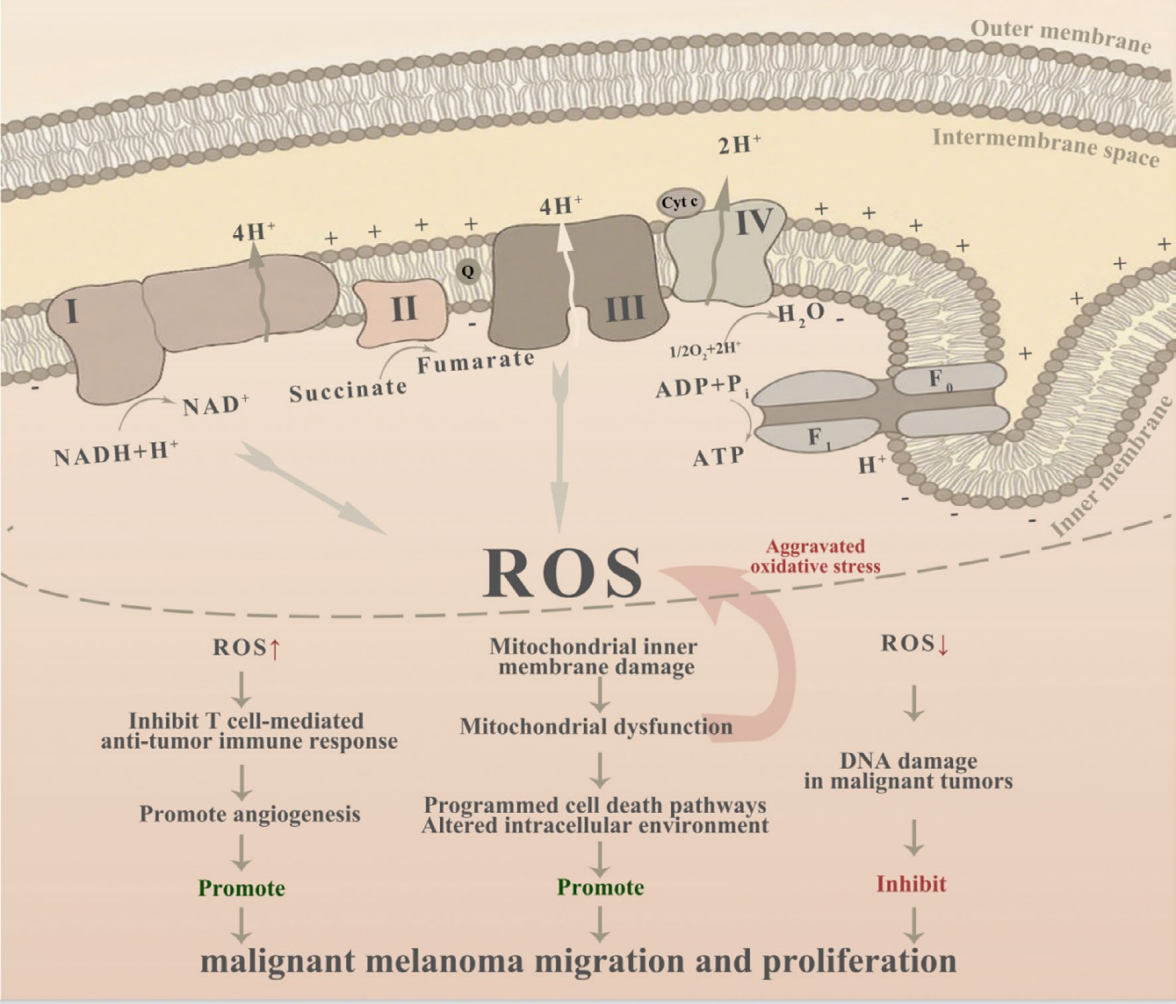
The dual role of reactive oxygen species in the development of melanoma, as well as the impact of mitochondrial dysfunction on antitumour immunity and tumour proliferation.

### 1.2 Definition and Role of Reactive Oxygen Species

Reactive oxygen species (ROS) are collectively defined as free radical and non‐radical oxygen‐containing species. Significant and major contributors of ROS in the cells are the mitochondria. Initially thought of as byproducts of cells, we now know that ROS have a dual function: cell signalling at low or modest concentrations, and oxidative damage to proteins, lipids and DNA at high concentrations [3, 4]. This type of oxidative stress has been implicated in a number of diseases such as cancer, neurodegenerative disorders and cardiovascular diseases. In the framework of the malignant process, the balance of ROS generation and removal is the key factor predetermining the occurrence and development of the disease process.

### 1.3 Importance of Mitochondria in Cellular Metabolism and Energy Production

Mitochondria are important in the cellular energy production via oxidative phosphorylation, in which ATP is constructed and endorses lipid and nucleic acid metabolism [5]. They also play a role in immune modulation, intracellular redox status, calcium homeostasis and ROS production, affecting a variety of signalling pathways. Mitochondrial dysfunction, which is associated with energy metabolism and ROS overproduction, has been shown to be involved in several diseases, including cancer, where it is seen to promote tumorigenesis and metastasis [6, 7]. A good apprehension of the mitochondrial functions in cellular metabolism is needed to devise solutions for malignant therapeutic interventions.

### 1.4 The Relationship Between ROS and Mitochondrial Dysfunction

ROS and mitochondrial dysfunction are closely connected and are the intertwined relationships. Mitochondria are a source of as well as a target of ROS generation, with generation occurring during ATP synthesis and mitochondria being vulnerable to oxidative damage [8]. The elevated ROS may compromise ATP generation, destabilise cell membrane potential and enhance the permeability of the mitochondria, eliciting apoptosis [9]. Mitochondrial dysfunction further increases ROS generation, and this culminates in a vicious cycle that increases the stress of the cells and leads to the progression of the disease. The interaction of these is of particular importance in malignancies, where oxidative stress is a central driver of tumorigenesis and an influence upon cancer cell behaviour [10]. Such mechanisms offer guidance on possible therapeutic interventions to oxidative stress‐related diseases, including malignancies.

## 2 Mechanisms of ROS Generation and Regulation

### 2.1 Sources of ROS in Mitochondria

Mitochondria are the organs that cause the most ROS formation in cells mostly through the electron transport chain (ETC) in activity, especially at Complexes I and III. Electron transfer may result in a reduction of oxygen, which is converted to superoxide radicals [11]. Such as probably mitochondrial dysfunction, elevated levels of membrane potential and metabolic substrates enhance ROS generation particularly by reverse electron transport (RET) [12]. Oxidative stress also worsens mitochondrial dysfunction, which initiates the process to build up the amount of ROS production and cellular damage in a feedback loop [13]. It is imperative that the maintenance of a balance between ROS production and scavenging is crucial because excessive levels of ROS result in oxidative stress which causes a variety of diseases [14]. Understanding these regulatory mechanisms, including those involving mitophagy and oxidative stress, is crucial for developing therapeutic approaches to mitigate oxidative damage in diseases like malignant melanoma [15].

### 2.2 Role of Intracellular Antioxidant Systems

Cells have elaborate antioxidant networks that significantly determine the concentration of ROS levels and alleviation of oxidative stress. Such systems are both enzymatic as well as non‐enzymatic in nature. Superoxide dismutase (SOD), catalase and glutathione peroxidase (GPX) belong to the key enzymatic antioxidants that interact to counteract ROS and avoid damage to cells [16]. Non‐enzymatic antioxidants, such as vitamin C, E, glutathione and other phytochemicals, also play an important role in the cellular defence against oxidative stress since they help to scavenge free radicals and metal ions that may catalyse the formation of ROS [17]. It is essential that these antioxidant defences be regulated. Nevertheless, due to pathological status, the antioxidant system may be overwhelmed. Oxidative stress leads to many diseases and pathological conditions, including cancer. Moreover, recent studies have shown that physical activity has a positive effect on stimulating these antioxidant systems, which further indicates that lifestyle modification could offer protection against oxidative stress [18, 19]. By clarifying the mechanisms in which these antioxidant systems act, researchers can obtain a target of improving cellular resistance to oxidative damage and health outcomes in general.

## 3 Biological Impacts of Mitochondrial Dysfunction

Mitochondrial dysfunction significantly disrupts cellular homeostasis and contributes to various diseases [20]. Its effects include alterations in energy metabolism and the regulation of programmed cell death pathways, both of which also impact the tumour microenvironment (TME). In cancer, these alterations have the potential to result in immune evasion or the inhibition of anticancer immune cell activity [21]. Programmed cell death (PCD), particularly apoptosis, necrosis, pyroptosis, ferroptosis and autophagy, is critical in establishing the immunosuppressive TME and clinical outcomes in cancer treatment [22]. Awareness of the biological effects of mitochondrial dysfunction is paramount to the development of counter strategies against its effects.

### 3.1 Changes in Energy Metabolism

Mitochondria play a pivotal role in cellular energy metabolism through oxidative phosphorylation. Mitochondrial dysfunction disrupts the electron transport chain, thereby impeding ATP synthesis and inducing energy deficits in tissues and organs [23]. Chronic mitochondrial dysfunction, especially that due to lipid overload, augments oxidative stress because of excess electron leakage and consequent ROS generation [24]. As a case in point, in Alzheimer's disease, mitochondrial dysfunction increases ROS production, contributing to neuronal damage and cognitive decline [25]. On the same note, failure in mitochondria in systemic diseases, such as heart failure, highlights its importance in metabolic wellness [26]. In malignant melanoma, mitochondrial dysfunction sustains the survival and an increase in tumour cells, confirming the importance of this process in cancer development. Therapeutic implication of the mechanism examined herein has the potential to be used to treat malignant.

### 3.2 Activation of Apoptotic Pathways

The association of mitochondrial dysfunction with the process of apoptotic pathways activation results in the pattern of programmed cell death. The intrinsic pathway of apoptosis is extremely reliant on the integrity of the mitochondrion because it comprises the discharge of the important pro‐apoptotic factors including cytochrome c into the cytosol. This release initiates the caspase‐activation cascade, reaching cell death [27]. In response to stressors like oxidative damage or energy depletion, dysfunctional mitochondria can initiate apoptosis by releasing these apoptogenic factors. In glioma cells, it has been proven to trigger apoptosis through the mitochondrial apoptotic pathway, which shows that the healthiness of mitochondria is an important indicator of cell survival [28]. Also, the cGAS‐STING pathway that is triggered by the cytosolic DNA is a potential signal that causes regulation of apoptosis via mitochondria. This points to the complex interaction between aspects of mitochondrial function and cell death pathways [29]. Through its activation of the cGAS‐STING signalling pathway, it does not only disrupt cell integrity but it also adds to advancing diseases related to chronic inflammation and tissue degeneration [30]. Meanwhile, it has also been illustrated in the literature that cGAS‐STING can have a significant effect on antitumour immune response in malignant melanoma [31]. With the correlation between apoptosis and mitochondrial dysfunction, the induction of apoptotic pathways plays a significant role in terms of the progress of malignant melanoma.

## 4 Dual Role of ROS in Malignant Melanoma

### 4.1 Promoting Tumour Cell Proliferation and Metastasis

ROS are major intermediaries in the development of malignancies, which help in the growth and spread of cancer cells. An increase in ROS in the tumour microenvironment is noted to promote the aggressive phenotype of the cancer. ROS stimulate several signalling pathways that confer increased cell proliferation and survival. As an example, the hepatocyte growth factor (HGF)/c‐Met signalling axis has been found to induce ROS production to stimulate epithelial‐mesenchymal transition (EMT) and metastasis in malignant melanoma cells [32]. Interestingly, BRAF inhibitors are widely used in the treatment of malignant melanoma, which act to increase ROS levels and boost metastatic activities [33]. On the contrary, other molecules, including salidroside, act as inducers of mitochondrial dysfunction and ROS‐mediated ferroptosis, and in effect, inhibit malignant cell growth and migration [34]. These data highlight the multifaceted role of ROS in malignant melanoma, acting simultaneously as signal transducers stimulating tumourigenesis and as effectors of cell death under specific conditions.

### 4.2 Apoptotic Induction and Antitumor Activity

ROS can also indispensably induce apoptosis and antitumor effects in malignant melanoma. The capacity of natural compounds to elevate the level of ROS and induce apoptosis of malignant melanoma cells has already been proved in many studies. As an example, naringenin, a flavonoid compound present in citrus fruits, exhibited an ability to reduce the viability of malignant melanoma cells eliciting production of ROS, which damaged the mitochondrion and triggered pro‐apoptotic signalling pathways [35]. Equally, the phenolic compound of olive oil, namely hydroxytyrosol, has a significant effect that increases ROS levels, triggers apoptosis and inhibits cell proliferation in malignant melanoma cells [36]. Further, sonodynamic treatment (SDT) has demonstrated potential in maximising apoptosis in malignant melanoma cells with the use of ultrasound to promote the generation of ROS [37]. Although ROS drives tumour expansion and metastasis, they are potent inducers of apoptosis; thus, a two‐pronged approach of targeting ROS to treat malignant melanoma is needed. The challenge is in the select choices of cracking the pro‐apoptotic effects of ROS and reducing the tumour‐promoting effects of ROS (Figure 1).

## 5 Regulation of ROS in the Microenvironment of Malignant Melanoma

### 5.1 Influence of Tumour‐Associated Macrophages

Tumour‐associated macrophages (TAMs) are a key factor of the tumour microenvironment (TME), which show phenotypic plasticity differentiating into either pro‐inflammatory M1 macrophages or immunosuppressive M2 macrophages [38]. M1 macrophages play an essential role in the eradication of tumour cells through antibody‐dependent cellular cytotoxicity, whereas M2 macrophages impair the antitumour immune response of T cells and stimulate angiogenesis, promote tumour growth and metastasis [39]. Recent reports propose that TAMs are the mediators of ROS within the TME, which may play conflicting roles in cancer biology. On the one hand, a high level of ROS may also cause oxidative stress, damage and programmed cell death of cancer cells. Conversely, excessive ROS can assist in the survival and metastasis of tumour cells by increasing angiogenesis and evasion of immune responses [40, 41].

The communication between malignant melanoma cells and tumour‐associated macrophages (TAMs) is bidirectional, with malignant melanoma cells inducing behavioural changes in TAMs, typically inducing M2 polarisation. This transition is mediated by cytokines and exosomes from malignant melanoma cells, which modulate TAM activity and generate an immunosuppressive tumour microenvironment favourable to tumour progression [42, 43]. Furthermore, metabolic reprogramming of TAMs in the cancerous microenvironment increases their pro‐tumour effects and plays a role in resistance to treatment [43]. It is imperative to understand the complex interaction of TAMs, ROS levels and malignant progression to arrive at therapies that can disrupt this vicious cycle.

### 5.2 Regulation of Intercellular Signalling

The intercellular communication in the malignant melanoma microenvironment is instrumental in dictating tumour behaviour and responding to therapeutic interventions. Tumour cells communicate with stromal and immune cells, including TAMs, by means of complex signalling that controls the levels of ROS and affects several key processes, including proliferation, apoptosis, and immune evasion. In one example, intracellular ROS in medium‐high abundance of TAMs can mediate tumour aggressiveness by enhancing TNF‐alpha (TNF‐alpha) production by TAMs [44].

Also, malignant and TAM cells communicate through secreting small extracellular vesicles (sEVs) that contain bioactive miRNA and proteins. These vesicles mediate changes in recipient cell behaviours, and these alter changes in factors that promote tumour survival and growth [45]. In addition, the hypoxic microenvironment of malignant cells downregulates the melanocyte markers and enhances invasion potential via a HIF‐1 alpha‐mediated mechanism. The mechanism is that the mitochondrial ROS drive the process of stabilising hypoxia‐inducible factor 1‐alpha (HIF‐1α), thus activating the proto‐oncogene Met that fuels malignant microenvironment metastasis [46]. Elucidating these intercellular signalling processes is critical to devising ways of effectively attacking the malignant microenvironment and thereby combining treatment responses.

## 6 Potential Therapeutic Targets and Prospects

### 6.1 Application of Antioxidants

The therapeutic application of antioxidants has gained considerable popularity because of the possible benefits in terms of millions of diseases caused by oxidative stress. Antioxidants make the ROS or reactive oxygen species neutral, as they are harmful when their quantities are in excess and are known to cause cellular damage, inflammation, and apoptosis. Recent reports focus on the antioxidants in dentistry; the treatments increase the oxidative stress due to the production of ROS. For example, the administration of exogenous antioxidants during dental surgery to minimise oxidative damage by patient treatment has been suggested to improve patient outcomes and moderate complications, including periodontitis and pulpitis [47]. Moreover, antioxidant nanozymes are emerging as a promising solution because, with higher stability and more cost‐effectiveness than natural enzymes, they are able to scavenge ROS efficiently by means of enzyme‐like activity [48]. Phenolic compounds are well known to have antioxidant properties and are highly prevalent in many foods and have great health benefits, such as cardioprotective [49]. Antioxidants play a role in cancer therapy too. Dietary intake of flavonoids, a group of C15 polyphenols, inhibits cancer progression and promotes DNA repair due to a p53‐dependent mechanism of antioxidant activity in human cells [50]. Flavonoids have also been found to play a role as antioxidants, decreasing ROS levels and inhibiting the pro‐tumorigenic functions of NF‐κB to delay tumour progression [51]. Lignans have shown antitumor activity against hormone‐dependent breast and prostate cancers and colorectal cancer [52], with antioxidant and anti‐inflammatory effects. As the volume of research expands, the integration of antioxidants into clinical practice appears to be a prospective strategy for addressing disorders associated with oxidative stress and for enhancing outcomes across various diseases.

### 6.2 Therapeutic Strategies Targeting Mitochondrial Function

Due to the essential function in cellular metabolism and energy generation, mitochondria are implicated in numerous diseases, particularly neurodegenerative disorders. Treating mitochondrial function is one of the digestive therapies that has shown promise. Mitochondrial autophagy, a crucial mechanism for maintaining cellular homeostasis and preventing diseases, has been applied in the treatment of diseases such as Parkinson's disease [53, 54]. Likewise, therapies focusing on the mitochondrial system have shown great promise in the treatment of the malignant melanoma.

For example, combining BCL‐xL inhibitor (BCLi), a mitochondrial protein inhibitor, with alisertib (AURKai), an emerging cancer treatment, induces malignant melanoma cell senescence when used as a monotherapy [55]. Moreover, a newly designed nanodrug (CDDP‐CS‐CYS‐EA, CCEA) using chondroitin sulfate (CS), anti‐angiogenic peptide (endostatin2‐alft1, EA) and cisplatin (CDDP) demonstrated the ability to induce apoptosis of malignant melanoma cells. This is accomplished through an increase in the expression of Bax, a decrease in Bcl‐2, an increase in mitochondrial membrane potential, the release of cytochrome C (Cyto C), as well as the increase in caspase 9 and caspase 3 activities [56].

Further, mitochondria‐targeted drugs enhance the efficiency of target therapy, bypassing the dynamic between fast‐ and slow‐cycling tumour cells. These medications also increase sensitivity to immunotherapy by immunomodulating the malignant microenvironment [57]. In totality, therapeutic strategies directed at mitochondrial action represent a multifaceted approach, which has a high potential to offer treatment for diseases that manifest themselves through mitochondrial dysfunction.

In conclusion, the role of ROS in mediating mitochondrial dysfunction is critically important in the progression of malignant melanoma. This interaction not only has provided insight into the mechanisms that underpin malignant melanoma pathophysiology but also makes plain to the dual nature of ROS. Traditionally considered detrimental, ROS are noted to play an important role in cellular signalling and adaptation, complicating their interpretation in tumour biology.

Investigation of the interrelation between ROS and the functioning of mitochondria provides potential directions for new treatment approaches in malignant melanoma. Explaining the relationship between ROS formation, mitochondrial health and malignant changes, researchers will be able to identify biomarkers and targeting treatment points that can enhance the effectiveness of treatment. Agents with the capacity to modulate reactive oxygen species (ROS) levels or restore mitochondrial function may play a crucial role in surmounting drug resistance, which persists as a formidable challenge in the treatment of malignant melanoma.

In future studies, the focus should be directing the findings of various studies that are contradicting, since these studies tend to have conflicting views on the role of ROS in cancer. The balanced view, advocating the beneficial and the adverse role of ROS, will ultimately make the difference in the malignant treatment. Individually pre‐planned interventive approaches that are designed to meet specific variations of levels in ROS and mitochondrial activity have great promise to enhance patient survival rates.

Moving forward, collaborative work across disciplines will be essential to leverage research knowledge on ROS and mitochondrial research. Such undertakings can result in the identification of new drug leads and new therapeutic enhancements of established drugs, thus opening a new treatment paradigm in malignant melanoma.

## Author Contributions

Lei Li: writing – review and editing. Zhiwei Zeng, Tengxiao Ma: conceptualization, writing – original draft. Bo Hu, Muye Guo, Qiaoxing Wang: research and collation of papers and literature.

## Disclosure

We confirm that all figures are our original work and have not been published nor are they under consideration for publication elsewhere.

## Ethics Statement

Ethical approval was not required as this study is based exclusively on published literature.

## Conflicts of Interest

The authors declare no conflicts of interest.

## Data Availability

The authors have nothing to report.
